# Pathological structure of visuospatial neglect: A comprehensive multivariate analysis of spatial and non-spatial aspects

**DOI:** 10.1016/j.isci.2021.102316

**Published:** 2021-03-16

**Authors:** Yusaku Takamura, Shintaro Fujii, Satoko Ohmatsu, Shu Morioka, Noritaka Kawashima

**Affiliations:** 1 Department of Rehabilitation for the Movement Functions, Research Institute of National Rehabilitation Center for Persons with Disabilities, 4-1 Namiki, Tokorozawa, Saitama, Japan; 2 Graduate School of Health Science, Kio University, Nara, Japan; 3 Nishiyamato Rehabilitation Hospital, Nara, Japan; 4 Neurorehabilitation Research Center, Kio University, Nara, Japan

**Keywords:** Medical Imaging, Behavioral Neuroscience, Systems Neuroscience, Computer Modeling

## Abstract

Visuospatial neglect (VSN) is a neurological syndrome of higher brain functions in which an individual fails to detect stimuli on a space that is contralateral to a hemispheric lesion. We performed a comprehensive multivariate analysis based on the principal component analysis (PCA) and cluster analysis in patients with right hemisphere stroke and then performed a determination of different elements of VSN. PCA-based cluster analysis detected distinct aspects of VSN as follows: cluster 1: low arousal and attention state, cluster 2: exogenous neglect, cluster 3: spatial working memory (SWM) deficit. Lesion analysis revealed neural correlates for each cluster and highlighted “disturbance of the ventral attention network” for the stagnation of exogenous attention and “parietal damage” for SWM deficit. Our results reveal a pathological structure of VSN as multiple components of an attention network deficit, and they contribute to the understanding of the mechanisms underlying VSN.

## Highlights

• This study attempted to establish the pathological structure of visuospatial neglect
• PCA revealed four distinct fundamental components underlying visuospatial neglect
• GMM-based clustering detected six subtypes of visuospatial attention network deficit

## Graphical abstract

**Figure:**
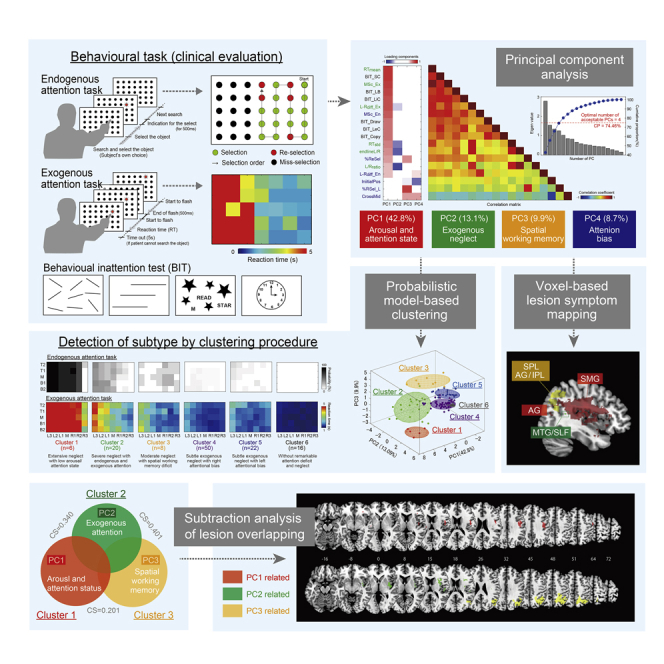

## Introduction

Visuospatial processing is a fundamental aspect of human cognitive behavior. Neurophysiological studies have attempted to reveal the nature of the neural mechanisms underlying visuospatial attention and have yielded insights into the global structure of neural networks involved in multi-modal and a wide range of components of neural processing. In 2002, Corbetta and Shulman (Corbetta and Shulman 2002) identified two distinct forms of attentional pathway: one is a dorsal pathway, with connections between the superior parietal lobule (SPL)/the intraparietal sulcus and the frontal eye field, and the other is a ventral pathway, with connections between the temporo-parietal junction and the middle frontal gyri (MFG) and inferior frontal gyri (IFG). The dorsal pathway is known as the dorsal attention network (DAN), which is associated mainly with goal-directed selection (Corbetta and Shulman., 2002; Gitelman et al., 1999; Kim and Cave, 1999; Rosen et al., 1999; Hopfinger et al., 2000; Beauchamp et al., 2001). The ventral pathway is known as the ventral attention network (VAN), and it is suspected to mediate shifts of attention when triggered by unattended or unexpected stimuli. From the functional point of view, the DAN is a high-level form of “top-down” endogenous attention to locations or features, and the VAN is a low-level form of “bottom-up” attention which is elicited by exogenous factors. A persuasive methodology to clarify the mechanisms that underlie the visuospatial attention network is the investigation of causal relationships between neurological symptoms and specific brain lesions. For example, the pathological feature of spatial neglect due to right hemisphere damage could indicate a causal relationship between damaged brain areas and neurological symptoms, as described by Pizzamiglio and his colleagues (Bisiach et al., 1996; Guariglia et al., 1993; Robertson et al., 1997). Studies of patients with visuospatial neglect (VSN) have also shed light on the network of brain areas that may be involved in normal spatial cognition and attention, based on the lesions that are typical in neglect. Neglect symptom was classically regarded as a parietal syndrome, but it has become evident as a widespread attention network disorder (Corbetta and Shulman., 2011; Doricchi et al., 2008; Verdon et al., 2010; Karnath and Rorden, 2012; Bartolomeo et al., 2012). While distinct components of VSN have been identified, such as sustained attention deficit (Robertson et al., 1997; Karnath 1988; Rengachary et al., 2011), spatial working memory (SWM) deficit (Malhotra et al., 2009; Husain et al., 2001; Toba et al., 2018), magnetic attraction (Toba et al., 2018; Saj et al., 2018), and perceptive/visuo-spatial, exploratory/visuo-motor, and allocentric/object-centered aspects of neglect (Verdon et al., 2010), VSN can be regarded as a multi-component and the wide range attention network disorder. It is evident that a wide range of symptomatic heterogeneity in patients with VSN can be largely attributed to multi-component deficits including the widespread visuospatial attention network. Husain (2019) recently described that none on their own is likely to lead to the full-blown syndrome. Different individuals might have different combinations of deficits depending upon the extent of their brain lesion.

In the present study, we evaluated three elements of VSN in 122 patients who had suffered a right hemisphere stroke: sustained attention, exogenous/endogenous attention, and SWM. Based on a variety of evaluation parameters, we first performed a principal component analysis (PCA) of selected variables obtained across three tests. PCA has been recently utilized to reveal individual's neurological behavior into main component (Corbetta et al., 2015). This analysis process was designed to break down neglect symptom into coherent profiles of co-varying deficits; the PCA results provided four extracted principal components (PCs) that explain 74.46% of the total variance. We then performed a Gaussian mixture model (GMM)-based clustering using four PC scores to discover the distinct type of VSN. As the result, we categorized six distinct clusters by conducting a multivariate analysis. Each cluster can be regarded as a “subtype” of VSN which consists of different contribution of the four PCs.

In order to confirm the above mentioned behavioral characterization, the brain areas responsible for symptomatic features were detected by using a subtraction analysis of brain lesions overlapping among the clusters and voxel-based lesion symptom mapping (VLSM) by the four detected PCs. The analyses revealed specific neural correlates for each of the symptomatic components, and they highlighted “disturbance of the VAN” for the stagnation of exogenous attention and “parietal area damage” for SWM deficit. Another important finding was that the patients' neurological symptoms and functional deficits after they suffered lesions were affected not only by disturbance of the attention network (which was lesion specific) but also by compensation for the symptoms/deficits over time. Our classification includes not only lesion-specific symptoms but also elements concerning compensation (i.e., intentional bias to neglected space). We provide the longitudinal data of four representative patients as part of a discussion of how symptoms change with the time course of recovery.

To the best of our knowledge, this is the first study to establish a pathological structure of VSN using comprehensive multivariate analyses based on various aspects of a clinical evaluation. Our results indicate that the clinical manifestations of VSN might reflect a combination of distinct components affecting different aspects of spatial and non-spatial symptoms.

## Results

**Figure 1:**
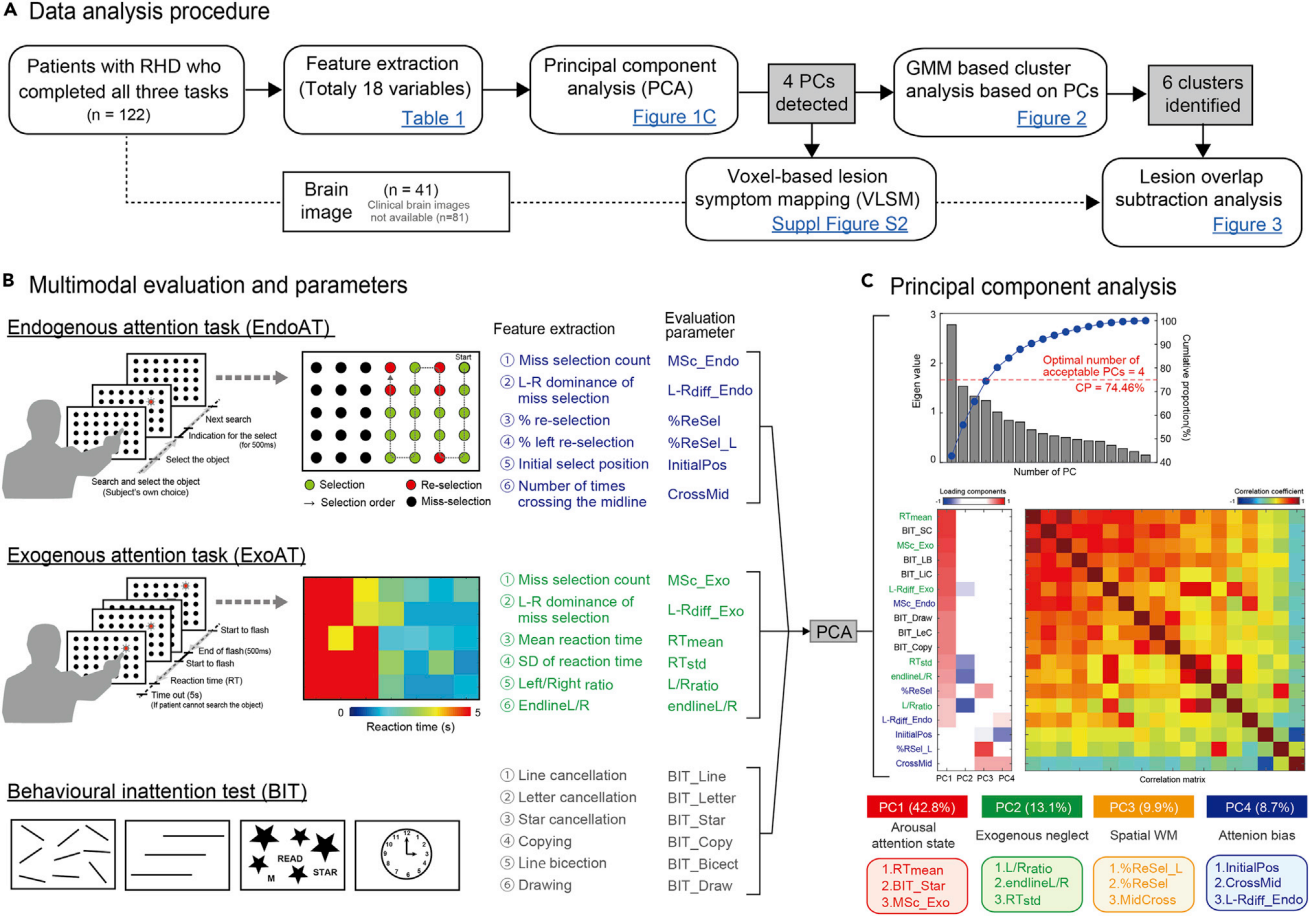
Framework of this study (A) Flow chart of data analysis procedure in this study. (B) Evaluation task and variables for visuospatial neglect. All patients performed touch-panel personal computer (PC)-based choice reaction task and completed the Behavioral Inattention Test (BIT). The endogenous attention task (EndoAT) on the PC was administered to evaluate the patient's endogenous attention spatial working memory (SWM) and selection strategy. The patient was asked to choose all targets in any order. The exogenous attention task (ExoAT) was administered to evaluate the patient's exogenous attention and sustained attention. The patients were instructed to choose a randomly flashed target, and we then calculated the evaluated parameters based on the spatial distribution of the patients' reaction times. The BIT consisted of a line cancellation test, letter cancellation test, star cancellation test, copying test, drawing test, and line bisection test. Using the results of these three evaluation tasks, we quantified six variables in a subsequent analysis. (C) Principal component analysis (PCA). Top: Eigen value (loadings) of each principal components (PCs) (bar) and cumulative proportion (line) calculated by the PCA for dimensional reduction from 18 quantified variables. Middle: Results of loading values to the four extracted PCs and the correlation matrix between all variables. Each PC could be reasonably interpreted based on previous studies' findings as follows. PC1 was interpreted as arousal and attention state including sustained attention, exploratory neglect, and severity of neglect. PC2: neglect with exogenous attention. PC3: SWM and selection order. PC4: attention bias and selection order.

A total of 122 patients were enrolled in this cross-sectional retrospective study. Figure 1A shows the analytical procedures used in this study. All the patients performed a touch-panel choice reaction task on a personal computer's monitor, and they completed the Behavioral Inattention Test (BIT; Wilson et al., 1987). The touch-panel choice reaction task consisted of an endogenous attention task (EndoAT) and an exogenous attention task (ExoAT). The EndoAT was used for the evaluation of the patients' endogenous attention, SWM, and selection strategy (see Figure 1A and Table 1). With the patient's results on the three evaluation tasks (i.e., the EndoAT, ExoAT, and BIT), we quantified each of the six variables for the subsequent analysis. We then subjected the data to the following three adjustments for the subsequent exploratory/data-driven analysis: (a) dimensional reduction from 18 variables using PCA to elucidate the neglect-related deficit components; (b) GMM-based probabilistic clustering using the four obtained PCs to elucidate different combinations of neglect-related components for each patient; and (c) lesion overlap subtraction of each cluster and VLSM for the four obtained PCs in order to understand the neural mechanisms underlying the neglect-related symptomatic components.

**Table 1:** Characteristics of each variable

	Variable	Task	Calculation	Range	Meaning
1	MSc_En	EndoAT	Total targets—count of total selection	0–35	Total capacity for endogenous attention.
2	L-Rdiff_En	EndoAT	MSc_En in left hemispace – MSc_En in right hemispace	−15–15	Neglect in endogenous attention: L-Rdiff_En = 0 indicates no difference between the left and right spaces.
3	%ReSel	EndoAT	Count of reselection/count of total selection ∗ 100	0–100	Spatial working memory: %ReSel = 100% indicates the reselection of all targets.
4	%ReSel_L	EndoAT	Count of reselection in left space/count of total selection in left space ∗ 100	0–100	Spatial working memory in left space: %ReSel_L = 100 indicates reselection of all left targets.
5	InitialPos	EndoAT	Initially selected position (the left top target is 1 and the right bottom target is 35.)	1–35	Exploration strategy.
6	CrossMid	EndoAT	No. of midline crossings (excluding RS target)	Inf	Exploration strategy.
7	MSc_Ex	ExoAT	No. of targets—count of total selection	0–35	Total ability of exogenous attention.
8	L-Rdiff_Ex	ExoAT	MSc_Ex in left hemispace – MSc_Ex in right hemispace	−15–15	Neglect in exogenous attention: L-Rdiff_Ex = 0 indicates no difference between left and right space.
9	RTmean	ExoAT	Mean reaction time	Inf	Sustained attention.
10	RTstd	ExoAT	Standard deviation of reaction time	Inf	Variability of attention, sustained attention
11	L/Rratio	ExoAT	Mean reaction time in the left hemispace/Mean reaction time in the right hemispace	Inf	Neglect in exogenous attention: L/Rratio = 1 indicates no difference between left and right space.
12	EndlineL/R	ExoAT	Mean reaction time in the left end column/Mean reaction time in the right end column	Inf	Neglect in exogenous attention: EndlineL/R = 1 indicates no difference between left and right end line.
13	BIT_Line	BIT	Count of total cancellation (conventional evaluation method)	0–36	Score of the line cancellation test in BIT: Explorational neglect.
14	BIT_Letter	BIT	Count of total cancellation (conventional evaluation method)	0–40	Score of the letter cancellation test in BIT: Explorational neglect.
15	BIT_Star	BIT	Count of total cancellation (conventional evaluation method)	0–54	Score of the star cancellation test in BIT: Explorational neglect.
16	BIT_Copy	BIT	No. of complete (conventional evaluation method)	0–4	Score of the copying test in BIT: Visual neglect and construction.
17	BIT_Bicect	BIT	Deviation is divided into three score areas (conventional evaluation method)	0–9	Score of the line bisection test in BIT: Degree of deviation in neglect space.
18	BIT_Draw	BIT	No. of complete (conventional evaluation method)	0–3	Score of the drawing test in BIT: Visual neglect and construction.

### Principal component analysis

The top panel of Figure 1C shows the eigen value (loadings) of each PCs and cumulative proportion obtained by the PCA for the dimensional reduction from the 18 quantified variables. The optimal number of acceptable PCs based on two criteria is four PCs (see the methods). The middle panel of Figure 1C shows a result of the analysis of the loadings of the four acceptable PCs and the correlation matrix between all variables. The results show that the PCs each have a characteristic loading based on the correlation structure between the variables.

The PCs could be reasonably interpreted based on the previous findings as follows. PC1 was interpreted as arousal and attention state, including sustained attention, exploratory neglect, and severity of neglect. This component had large contributions from mean reaction time in ExoAT (RTmean), star cancellation test in BIT (BIT_SC), and mis-selection count in ExoAt (MSc_Ex). PC2 was interpreted as neglect with exogenous attention. This component had large contributions from the ratio of the reaction time in the left hemispace to that in the right hemispace in ExoAT (L/Rratio) and the standard deviation of the reaction time in ExoAT (RTstd). PC3 was interpreted as SWM deficit and selection order. This component had large contributions from mainly overall space and the left space reselection rate of EndoAT (%ReSel and &ReSel left) and the number of midline crossings in EndoAT (CrossMid). PC4 was interpreted as attention bias and selection order. This component had large contributions from mainly the initial selection position in EndoAT (InitialPos), MidCross, and difference of mis-selection count between the left and right hemispace in EndoAT (L-Rdiff_En). We used the PC scores for the detection of subtypes, symptom-specific brain areas with VLSM, and subtraction analysis via cluster analysis.

### Gaussian mixture model-based cluster analysis

**Figure 2:**
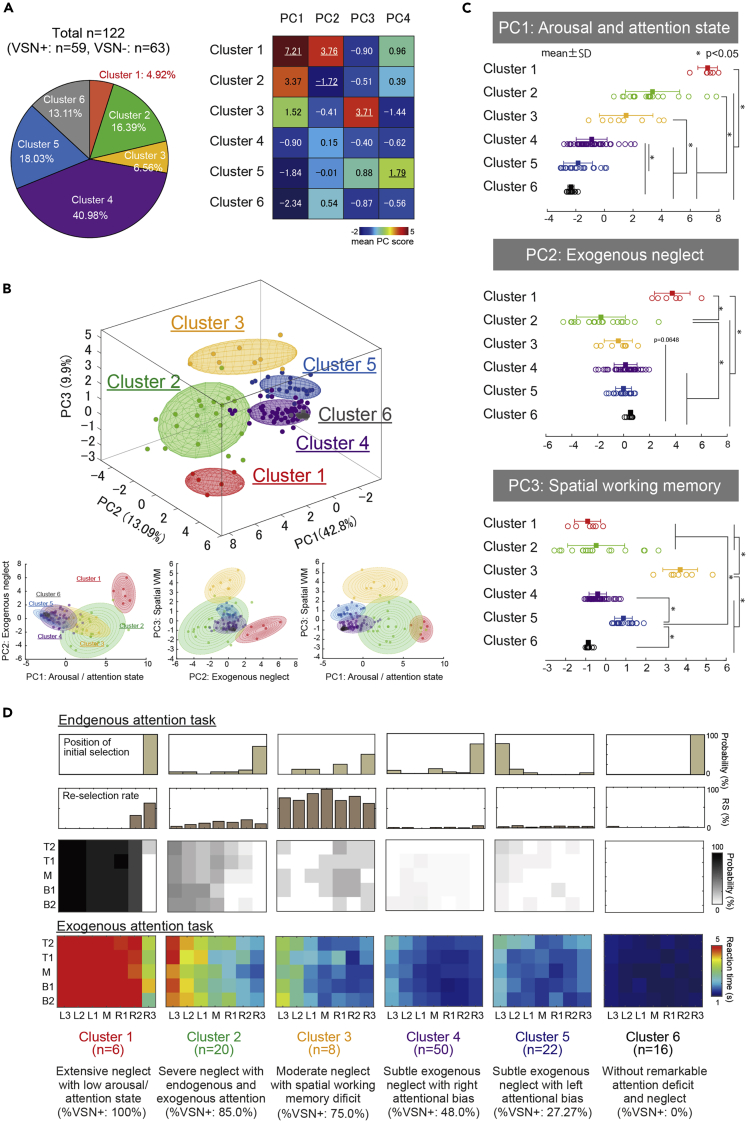
Summary of the Gaussian mixture model (GMM)-based cluster analysis (A) The percentages of each cluster's members are shown as a pie chart. Six patients (4.92%) were classified as cluster 1, 20 patients (16.39%) as cluster 2, eight (6.56%) as cluster 3, 50 (40.98%) as cluster 4, 22 (18.03%) as cluster 5, and 16 (13.11%) as cluster 6. The averaged PC scores in each cluster are presented as a color matrix. (B) Scatterplots of PC1 to PC3 in each cluster with 10%–90% confidence ellipsoids and ellipses. The three-dimensional plot and 90% confidence ellipsoids of PC1 to PC3 show the independence and characteristics of each cluster. (C) Results of the statistical comparison of the scores among clusters, shown separately for each of the main three PCs (top: PC1, middle: PC2, bottom: PC3) from Figure 1C. Data are represented as mean ± standard deviation (SD). Asterisks are represented as statistical significance (p < 0.05) to obtain by post hoc test (Steel-Dwass test) in Kruskal-Wallis test. (D) Summary of the EnAT and ExAT scores in each cluster. Top row: The proportion of the initial selection position in each cluster at the EnAT. Second row: The re-selection probability in each column. Third row: The spatial distribution of selection probability values in the EnAT. Fourth row: The spatial distribution of reaction times in the ExAT (red = slow reaction time).

To classify the subtypes of VSN, we used the PC scores for a GMM-based clustering. We selected the model showing high theoretical validity with a better Bayesian information criterion (BIC) and integrated complete-data likelihood (ICL) as the optimal model from among several models that met these criteria (see Table S1 for details). We detected six clusters from the 60 candidate models. The percentages of each cluster's members are shown as a pie chart in Figure 2A. The averaged PC scores in each cluster are presented as a color matrix in Figure 2A. Details of the basic attributes in each cluster are listed in Table 2.

**Table 2:** Difference in basic attribution between each cluster

Cluster	n	Age	Sex (F/M)	Time	MMSE	BIT	% VSN	CBS			
								n	Objective	Subjective	Diff
Cluster 1	6	79.5 (54–87)	1/5	33 (3–114)	16.5 (12–27)	26 (16–70)	100%	3	10 (6–15)	1 (1–3)	9 (5–12)
Cluster 2	20	69 (50–80)	7/13	57 (1–673)	22 (16–28)	103 (36–144)	85%	5	11 (10–15)	4 (0–7)	9 (3–11)
Cluster 3	8	68.5 (42–83)	2/6	47.5 (3–240)	23.5 (15–29)	123.5 (64–139)	75%	4	8 (5–9)	3 (1–4)	4 (4–7)
Cluster 4	50	65.5 (34–86)	18/32	46 (2–806)	25 (8–30)	131.5 (32–146)	48%	23	3 (0–15)	1 (0–12)	1 (−2–9)
Cluster 5	22	67 (33–90)	8/14	43.5 (4–265)	28 (13–30)	135.5 (115–146)	27.27%	9	0 (0–10)	0 (0–6)	0 (0–6)
Cluster 6	16	65 (42–86)	5/11	50.5 (4–1653)	29 (24–30)	144 (135–146)	0%	5	0 (0–1)	0	0 (0–1)
All	122	68 (33-90)	41/81	49 (1–1653)	25 (8–30)	131.5 (16–146)	48.36%	49	3 (0–15)	1 (0–12)	1 (-2–12)

The median (min-max) value is shown as a representative value for all variables. MMSE, mini mental state examination; BIT, Behavioral Inattention Test; % VSN, percentage of visuospatial neglect; CBS, Cathrine Bergego Scale; CBSd, difference of CBS.

Figure 2B is scatter plots of PC1 to PC3 in each cluster with 10%–90% confidence ellipsoids and ellipses. The three-dimensional plot and 90% confidence ellipsoids of PC1 to PC3 illustrate the independence and characteristics of each cluster. The PC1 and PC2 planes showed inverse U-shaped distributions, and each cluster is placed along clusters of high to low severity. In contrast, clusters 2 and 3 on the PC1 and PC3 planes are split in the domain of PC3. A statistical comparison of the scores among clusters was performed separately for each of the main three PCs in Figure 2C. The score of PC1 was significantly larger in cluster 1 than in the other clusters and significantly larger in clusters 2 and 3 than in clusters 4–6 and significantly larger in cluster 4 than in clusters 5 and 6 (χ2 = 84.726, df = 5, p = 8.59 × 10^−17^, r = 0.728). The score of PC2 was significantly smaller in cluster 2 compared to clusters 1, 4, 5, and 6 without cluster 3. Cluster 1 was significantly larger than the other clusters (χ2 = 43.505, df = 5, p = 2.919 × 10^−8^, r = 0.502). PC3 was significantly larger in cluster 2 than in the other clusters and significantly larger in cluster 5 than in the other clusters (χ2 = 69.113, df = 5, p = 1.568 × 10^−13^, r = 0.668). The detailed statistical results are provided in Table S2.

Figure 2D illustrates the summarized EndoAT and ExoAT results in each cluster, including the proportion of the initial selection position in each cluster at the EndoAT and the re-selection probability. The spatial distributions of selection probability in the EndoAT and the reaction times in ExoAT are illustrated. Each cluster shows different results for each evaluation point. Cluster 1 can be characterized by extensive neglect with a low arousal/attention state: namely, difficulty of selection in the EndoAT, delayed reaction time not only in the left space but also in the right space, and a high probability of re-selection only in the right space. Cluster 2 can be characterized by severe neglect with endogenous and exogenous attention, i.e., difficulty of selection in the left space in the EndoAT, and extensive delayed response in the left space at the ExoAT.

Cluster 3 can be characterized by moderate neglect with a deficit of SWM; a high probability of re-selections on both the left and right spaces and a moderate extent of delayed response in the left space at the ExoAT. Cluster 4 can be characterized by subtle exogenous neglect with right attentional bias, i.e., a subtle delayed response in the left space at the ExoAT with right attentional bias. Cluster 5 can be characterized by subtle exogenous neglect with left attentional bias: a subtle delayed response in the left space at ExoAT with left attentional bias. Cluster 6 can be characterized as without remarkable attention deficit and neglect, that is, better performance than other clusters in both tasks.

### Lesion subtraction analysis for two distinctive clusters

**Figure 3:**
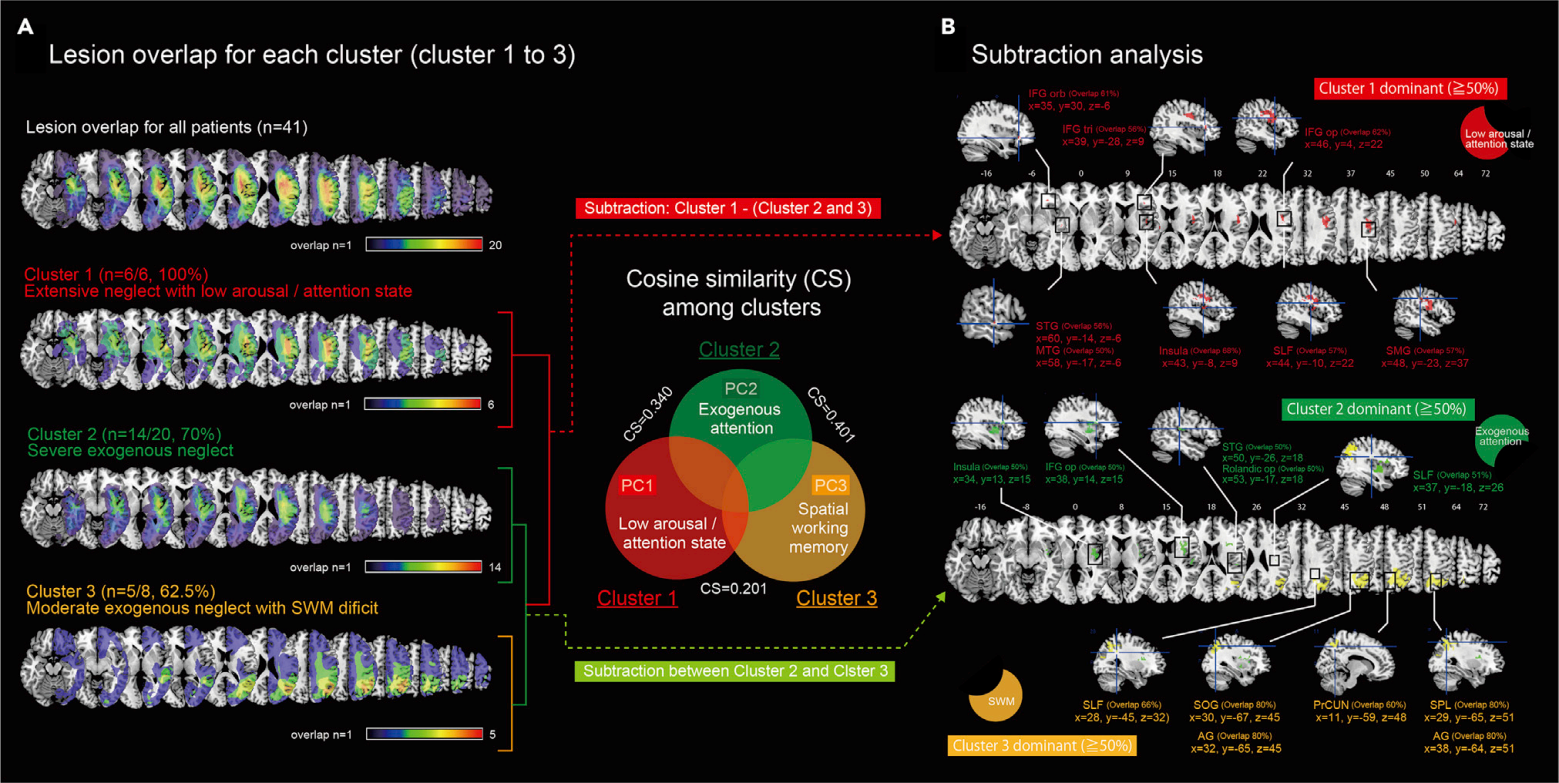
Lesion overlapping findings and the results of the subtraction analysis among clusters (A) Top panel shows lesion overlapping among all 122 patients (n = 41). Bottom three panels show overlapping of clusters 1, 2, and 3. The Venn diagram is a schematic representation of the similarities/differences among three PCs based on the pairwise cosine similarity (CS) distances among clusters. The distance between the center of each circle reflects the CS value. The difference of each cluster clearly reflects distinct aspects of visuospatial neglect as follows: [arousal and attention state = cluster 1\(cluster 2 ∪ cluster 3), exogenous neglect = cluster 2\cluster 3, SWM = cluster 3\cluster 2]. We used this characterization for the subtraction analysis of the brain lesion overlapping, and we obtained the specific neural correlates for each of these components. (B) Top: The detected lesion by a subtraction of cluster 1 from clusters 2 plus 3. The dominant lesion areas (≥50%) are color highlighted in each brain map. Bottom: The detected lesion by subtraction between cluster 2 and cluster 3. The detailed results of the subtraction analysis are summarized in Table S3.

Figure 3A shows the lesion overlapping among 41 of 122 patients. Overlap images of the clusters in which there were brain images of >50% of all cases in the respective cluster are provided in Figure 3A. The three clusters which were applicable had different features in the lesion area.

The Venn diagram in Figure 3A is a schematic representation of the similarities/differences among three PCs based on the pairwise cosine similarity (CS) distance among clusters. The difference of each cluster clearly reflects distinct aspects of VSN as follows: [low arousal and attention state = cluster 1\(cluster 2 ∪ cluster 3), exogenous neglect = cluster 2\cluster 3, SWM = cluster 3\cluster 2]. We used this characterization for the subtraction analysis of the brain lesion overlapping, and we then obtained specific neural correlates for each of these components. The lesions detected by subtracting cluster 1 from clusters 2 plus 3 are shown in Figure 3B. In consideration of the small number of subjects in each cluster and the difficulty of the statistical analysis, we set a criterion of at least 50% difference in overlapped area to minimize overestimation of the detected lesions (Lunven et al., 2015). The dominant lesions in cluster 1 were the insula (68%), the opercular part of the IFG (IFGop; 62%), the orbital part of the IFG (IFGorb; 61%), the superior longitudinal fasciculus (SLF: 57%), and the supramarginal gyrus (SMG: 57%). Figure 3B also shows the lesion detected by subtraction between cluster 2 and cluster 3. The dominant lesion areas in cluster 2 were mainly the insula (50%), the opercular part of IFG (IFGop; 50%), the superior temporal gyrus (STG: 50%), the opercular part of the Rolandic area (Rolandic op; 50%), and the SLF (51%). In contrast, the dominant detected lesions in cluster 3 were mainly the precuneus (PrCUN; 60%), the SLF (66%), the superiror occipital gyrus (SOG: 80%), the angular gyrus (AG: 80%), and the superior and inferior parietal lobules (SPL; 80%, IPL; 73%). The detailed results of the subtraction analysis are summarized in Table S3.

### Attention bias (domain of PC4)

**Figure 4:**
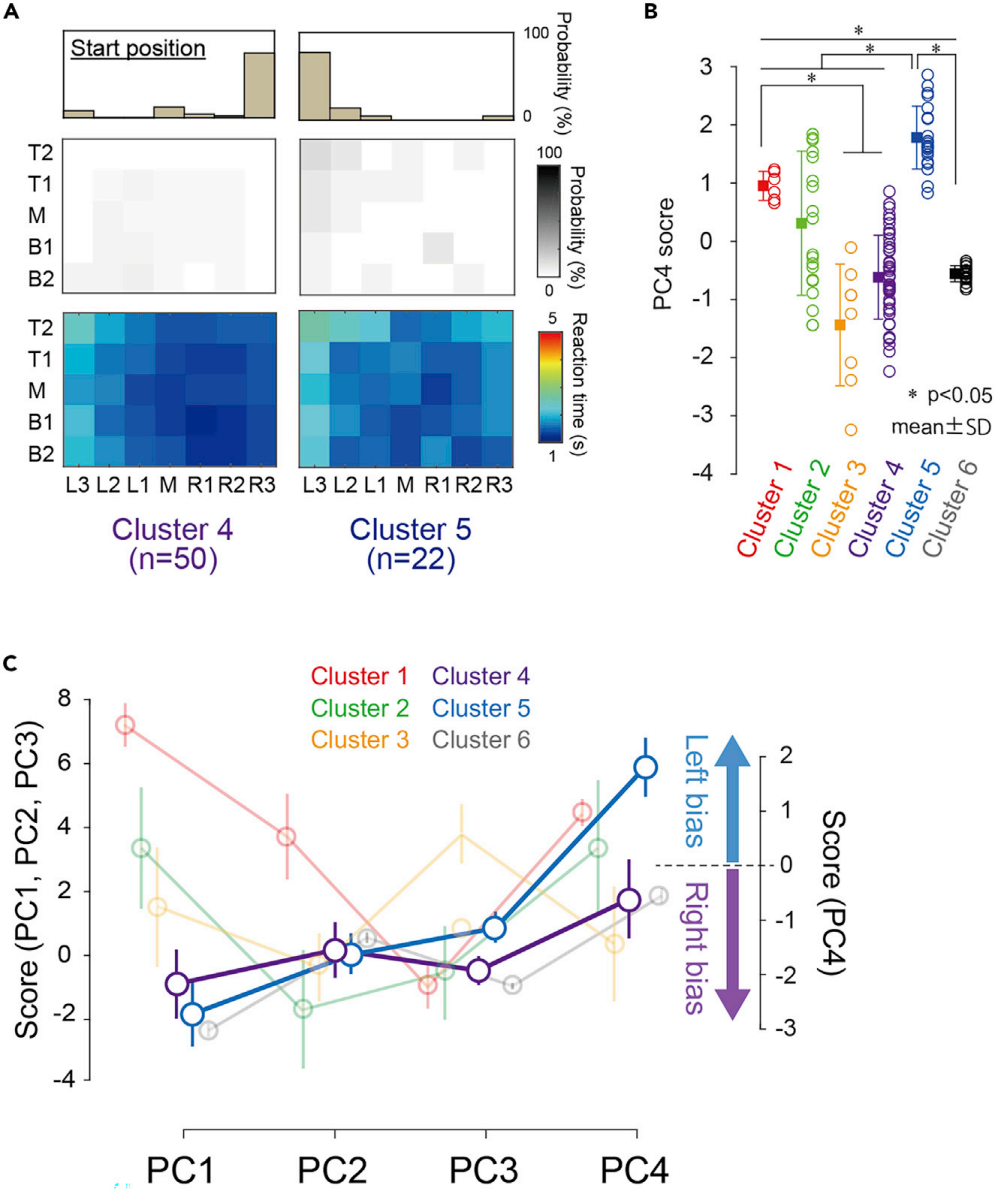
Component of attentional bias and selection strategy (A) Results of EnAT and ExAT in cluster 4 and 5. While the probability of selection and reaction time were identical, initial selection (start position) shows clear difference between two clusters. (B) Results of the statistical comparison of the scores among clusters, shown separately for each of the PC4. Data are represented as mean ± SD. Asterisks are represented as statistical significance (p < 0.05) to obtain by post hoc test (Steel-Dwass test) in Kruskal-Wallis test. (C) Summarized in averaged PC score in each clusters. The difference between cluster 4 and 5 was particularly clear in PC4. Data are represented as mean ± SD.

PC4 was interpreted as a component of attentional bias and selection strategy (Figure 1D). Figure 4A illustrates the summarized EndoAT and ExoAT results in cluster 4 and 5. Figure 4B showed the comparison of PC scores among clusters revealed that the score of PC4 was significantly larger in cluster 5 than in the other clusters (χ2 = 65.778, df = 5, p = 7.729 × 10^−13^, r = 0.649). The averaged PC scores in each cluster revealed that the difference between clusters 4 and 5 was particularly clear in PC4, whereas there was little difference in PC1, 2, and 3 (Figure 4C).

### Longitudinal analysis of four representative patients

**Figure 5:**
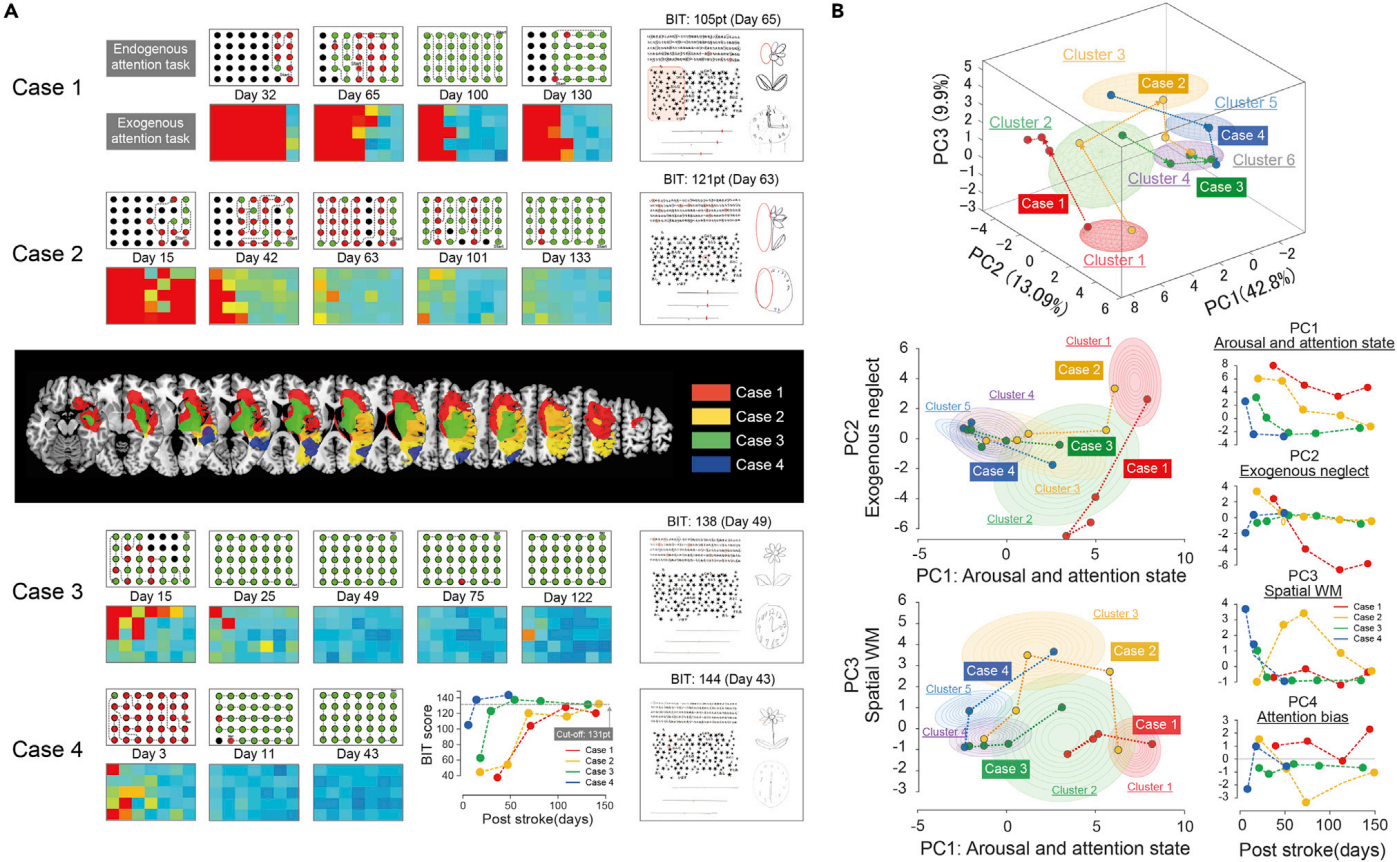
Longitudinal analysis in representative four patients (A) In four representative cases, the results of a pair of EnAT and ExAT and BIT score were demonstrated. Middle MRI image is overlapping lesion of four cases. Time course changes in total score of BIT were summarized in the bottom of Figure 5A. (B) Transition patterns among PCs in each case. This is a quantitative representation of the characteristic recovery process in representative cases (each case showed different traveling path). Contrast between case 1 and 2 seems to be clear that both cases originate from cluster 1 and then move to cluster 2 in case 1 (stagnation of exogenous attention) while move to cluster 3 in case 2 (SWM deficit). Contrast between case 3 and 4 reflects different aspect of recovery process, that is, both cases finally move to cluster 4, but originated from cluster 2 in case 3 (stagnation of exogenous attention) or from cluster 3 in case 4 (SWM deficit). These representative cases clearly demonstrated distinct modality of symptom and different recovery process and more importantly would give us materials to discuss mechanisms underlying visuospatial neglect and its pathological structure.

To test our interpretation of each PC and cluster, we observed the recovery process in four representative patients (Figure 5). At the first assessment, case 1 had much difficulty in both task (Figure 5A) and then gradually improved but was still stagnant EndoAT 130 days after stroke. Case 2 also had much difficulty at the first assessment but gradually improved his performance in both tasks, and he could select/respond all targets at 133 days. Patients 3 and 4 commonly showed difficulty of response only in the left space in the ExoAT, but their search behavior in the EndoAT showed a clear difference, i.e., patient 3 searched for the objects in a random order, and patient 4 showed many re-cancellations. Both patients tended to show good recovery within 1-month after stroke. The extent and characteristics of neglect behavior were also clearly reflected in the BIT results (Figure 5A). The time course changes in the patients' BIT total scores are summarized in Figure 5A.

The transition patterns among PCs in each case (Figure 5B) were obtained as a quantitative representation of the characteristic recovery process in the representative cases (each patient showed a different traveling path). The contrast between case 1 and 2 indicates that both cases originated in cluster 1 and then the patient moved to cluster 2 (stagnation of exogenous attention), whereas case 2 moved to cluster 3 (SWM deficit). The contrast between patients 3 and 4 reflects different aspects of the recovery process: both patients finally move to cluster 4, but case 3 originated in cluster 2 (exogenous neglect) and patient 4 originated in cluster 3 (SWM deficit). These representative cases clearly demonstrated a distinct modality of symptoms and different recovery processes, and more importantly, they provide findings that can be used to discuss the mechanisms that underlie VSN and its pathological structure.

## Discussion

It is evident that the wide range of symptomatic heterogeneity in patients with VSN is largely attributable to multi-component deficits including the widespread visuospatial attention network. We conducted the present study to establish the pathological structure of VSN by using a multivariate analysis and machine learning algorithms based on a variety of symptom-related evaluation parameters, and we sought to reveal the relationships between symptomatic features and the responsible brain areas. The results of the PCA revealed distinct four fundamental aspects of VSN: arousal and attention state, exogenous neglect, SWM, and attention bias. The GMM-based clustering detected six clusters that are reasonably characterized by the different contributions of the four PCs. These symptomatic characterizations were supported by the results of the subtraction analysis of overlapping lesions based on differences among the clusters that revealed specific neural correlates for each of these components. Moreover, the recovery process after unilateral spatial neglect (USN) was characterized by the transition from one cluster to another in accordance with the contribution of multiple components of VSN during each recovery period. Our results provide important point of view that the clinical manifestations of VSN might reflect a combination of distinct components affecting different aspects of spatial and non-spatial symptoms.

### Four distinct components of visuospatial neglect and the relevant lesions

There is no single or perfect clinical evaluation for assessing VSN. It is speculated that there are distinct subtypes or different cognitive components underlying neglect symptom so that it is unlikely that damage to a unique area could explain clinical manifestations. To reveal the neural mechanisms underlying VSN, it is necessary to correctly understand the pathological structure based on various aspects of symptomatic characteristics behind spatial neglect. In the present study, we evaluated different aspects of elements of VSN, i.e., sustained attention, exogenous attention, endogenous attention, and SWM based on several parameters, and we attempted to break down neglect symptom into coherent profiles of co-varying deficits. We detected four principal components, and these four PCs can be interpreted as follows: PC1: arousal and attention state including sustained attention (Robertson et al., 1997; Rengachary et al., 2011; Robertson et al., 1998; Husain and Rorden, 2003) and exploratory neglect (Verdon et al., 2010) and severity of neglect. PC2: neglect with exogenous attention (Corbetta and Shulman., 2002; Corbetta and Shulman., 2011; Doricchi et al., 2008; Rengachary et al., 2011; Husain and Rorden., 2003; Posner et al., 1984). PC3: SWM deficit (Malhotra et al., 2009; Husain et al., 2001; Toba et al., 2018). PC4: attention bias (Bartolomeo et al., 2001; Azouvi et al., 2002).

In order to identify the lesions responsible for the above mentioned PCs, we attempted voxel-based lesion symptom mapping (VLSM: see Figure S2). The detected areas of arousal and attention state-related PC1 were the whole area of VAN (slightly frontal). Exogenous neglect-related PC2 had relevant lesions in the STG, middle temporal gyrus (MTG), and SLF. SWM deficit-related PC3 had relevant lesions in the dorsal occipitotemporal area. PC4 had a unique nature which showed both positive and negative variability reflecting left and right attention bias, respectively. The detected areas of the PC4 left attention bias-related positive score were the middle occipital gyrus (MOG), AG, and SOG. The PC4 right attention bias-related negative score had a relevant lesion in the STG. Although these results were based on uncorrected statistical level, the detected lesions are in good agreement with the previous reports. We then attempted a further process for the characterization of VSN with the GMM-based cluster analysis, and we then attempted to estimate neural correlates based on a subtraction analysis of the detected subtypes.

### Six distinct subtypes of visuospatial neglect

With the PCA process, we were able to summarize the symptomatic nature of VSN as four principal components. Considering the wide heterogeneity, the nature of multiple components' deficits in VSN, we speculated that certain types of neglect consist of different contributions of each of the four components. We then attempted to detect the “subtypes” by conducting a GMM-based cluster analysis based on the PC score. GMM clustering is a probabilistic model-based clustering method and is more robust than other clustering methods (Banfield and Raftery 1993; Scrucca et al., 2016). The model with high theoretical validity with better BIC and ICL values was selected as the optimal model from among several models that met these criteria. As summarized in Figure 2, we detected distinct six clusters. Each cluster was clearly explained by the relative contributions of the four PCs. Cluster 1 was characterized by extensive neglect with a low arousal/attention state as reflected by a high PC1 score. This cluster also reflected the severity of visuospatial attention. Cluster 2 was characterized by severe neglect with endogenous and exogenous attention, as reflected by a lower PC2 score. Cluster 3 had an at least partly common characteristic with cluster 2 (a moderate extent of exogenous neglect), but a distinct symptomatic nature could be identified, i.e., a deficit of SWM reflected by a high probability of re-selections. The component of attention bias (PC4) was clearly reflected by differences between clusters 4 and 5, which had similar contributions to PC1, 2, and 3 but exhibited rightward or leftward attention bias, respectively (Figure 4). Concerning this point, it was suggested that the initial selection position is a sensitive indicator in determining neglect symptoms (Azouvi et al., 2002). Our previous study also revealed that in patients who recognized their own neglect behavior, the patients tended to pay attention toward the left neglected space (Takamura et al., 2016). It is likely that the left bias reflects a compensatory strategy.

### Neural correlates of visuospatial neglect

While the brain areas detected by VLSM were linearly correlated with a lesion with each component, those detected by the subtraction analysis can be regarded as specific lesions of each subtype. We observed the extent of common and different parts as quantified by cosine similarity. The Venn diagram in Figure 3 is a schematic representation of the differences among clusters 1, 2, and 3. We speculate that a portion of the difference in each cluster might reflect distinct aspects of VSN as follows: [low arousal and attention state = cluster 1\(cluster 2∪cluster 3), exogenous neglect = cluster 2\cluster 3, SWM = cluster 3\cluster 2]. We used this characterization for the subtraction analysis of brain lesion overlapping, and then, we revealed specific neural correlates for each of these components.

Cluster 1 was interpreted as a cluster showing stagnation of endogenous/exogenous attention with an arousal decrease (a delayed response was also observed in the right space). Independent lesion locations were concentrated mainly in the insula and IFG, which is reported as an important site of convergence for stimulus-driven and goal-directed attention (Asplund et al., 2010). Concerning this point, Rencgachary et al. showed that patients with spatial neglect accompanying damage to the IFG showed severe neglect behavior and a delayed response of detection and re-orientation toward the right space (Rengachary et al., 2011). Moreover, the right insula has been associated with sustained attention (Thakral and Slotnick, 2009) and its damage causes a decrease in the improvement effect of attention to the neglected space from an auditory warning (Chica et al., 2012).

While clusters 2 and 3 showed a similar aspect of exogenous neglect, each had distinct symptomatic neglect behavior, namely, cluster 2 showed severe stagnation of exogenous attention, and cluster 3 showed SWM deficits (many re-cancellations in the overall space). The subtraction analysis of lesion overlaps between those two clusters revealed distinct lesion sites. Cluster 2 has lesions in the insula, IFGop, STG, and anterior parts of the SLF, which correspond to the disturbance of the VAN and contribute to the occurrence of USN and its chronicity (Thiebaut de Schotten et al., 2005; (Karnath et al., 2011); Lunven et al., 2015). In contrast, cluster 3 had lesions at the AG, SPL, SOG, precuneus, and posterior parts of the SLF. Damage to the medial parietal cortex was reported to cause disorder of spatial navigation such as global disorientation (Boccia et al., 2014; Aguirre and D’Esposito, 1999) and body awareness disorder (Herbet et al., 2019; Wolpert et al., 1998). In addition, Cavanna and Trimble (Cavanna and Trimble., 2006) suggested that the precuneal cortex relates to self-related cognitive processes such as self-awareness, self-centered mental imagery strategies, social cognition, autobiographic memory, and others. Given these findings, it appears that the superior parietal area including the precuneal cortex is important for maintaining internal representations. Therefore, the symptom of cluster 3 can be characterized as an SWM deficit as a failure to maintain an internal representation for space between trans-saccadic updates. This interpretation is in good agreement with reports that damage in the parietal cortex is related to SWM (Malhotra et al., 2009; Husain et al., 2001; Toba et al., 2018).

The schematic representation with Venn diagram in Figure 3 was inspired by Figure 2 in Husain's commentary (Husain, 2019). As one of the main components consists of VSN, Husain clearly placed selective attention as direction bias in competition/in orienting attention. In our study, those components might have been involved in exogenous neglect and attention bias as revealed by PC2 and PC4, respectively. Through a comparison of results between VLSM and a subtraction analysis with the same patients, we identified an analysis procedure that can detect neural correlates behind VSN.

### General discussion based on recovery process in four representative cases

As shown in the Figure 5, the longitudinal data clearly demonstrated that each patient exhibited improvement of the neglect symptom over time, but the symptomatic nature and the time course (i.e., the transition from one cluster to another) differed among the patients. Such time-dependent changes in the symptomatic features were clearly characterized as transitions from one cluster to another, suggesting that some symptomatic characteristics could not be attributed to specific lesions.

Case 1, who showed clear stagnation of exogenous attention and remarkable left space neglect in BIT, could be regarded as having a higher probability of chronicity of VSN due to the disturbance of VAN (Thiebaut de Schotten et al., 2005; Karnath et al., 2011; Lunven et al., 2015). Case 2 had damage to the VAN and parietal cortex that may have caused neglect symptoms due to a failure of different components of spatial attention. The contrast between case 1 and 2 is a clear example of the distinct characteristics/subtypes of severe VSN. Interestingly, the difference in the contrast-recovery time course between cases 1 and 2 was clearly characterized as a form of transition among clusters. Case 3 and 4 are another pair showing different attention bias during their recovery. Both cases had a delayed reaction time on the ExoAT, but their behavior during the EndoAT was totally different: Case 3 lacked arousal and attention state as reflected by miss cancellations while case 4 showed extensive re-cancellation. Such different symptomatic natures clearly reflect the process of recovery in each patient. While case 3 transited from cluster 2 to 4 as a type of rightward bias due to attention deficit, case 4 transited from cluster 3 to 5 as a type of compensatory leftward attention. Although they had a disturbance of VSN in the acute phase, both patients showed good recovery at 45 days after the onset of stroke and finally completed both the EndoAT and ExoAT without remarkable errors, and their BIT scores far exceeded the cutoff at 49 days in case 3 and 43 days in case 4. The contrast between case 3 and 4 is a clear example of distinct characteristics/subtypes of mild VSN.

As clearly shown in Figure 5B, manifestations of neglect symptom change with time. Moreover, as reflected in attention bias (PC4), the recovery process involves not only lesion-specific symptoms but also a compensatory strategy. Although several studies described the time course of the recovery from neglect symptom (Ramsey et al., 2016; Nijboer et al., 2013), no previous study had revealed the recovery process based on the characterization of VSN. The study is the first attempt to demonstrate the recovery process based on a comprehensive analysis of neglect symptom. Our results provide important information about the clinical manifestations of VSN that might reflect a combination of distinct components affecting different aspects of spatial and non-spatial symptoms. We therefore conclude that the process of a PCA and a PC score-based cluster analysis suitably classifies subtypes; in other words, the present results revealed the pathological structure of VSN.

### Limitations and future direction

Further research is necessary to overcome the following limitations in the present study. First, we used only the overall score of the BIT. The results of paper-and-pencil tests essentially include the more detailed features of neglect symptom, e.g., the difference in the number of cancellations between the left and right in the cancellation task (Azouvi et al., 2002; Verdon et al., 2010), the characteristics of the copying task (Gainotti and Tiacci., 1970; Seki and Ishiai., 1996), a left hyperschemia (Rode et al., 2014), and others. Integrating these features may provide a more diverse and robust understanding of neglect symptoms. Second, in order to establish the pathological structure of VSN, we should attempt to establish a pathological model with structure estimated modeling with larger patient samples. Third, it is necessary to pay attention to the recovery process in order to establish a pathological model and responsible lesion for the symptomatic feature of VSN because a patient transits from one cluster to another during recovery as we showed in Figure 5. Most of the previous studies applied inclusion criteria of the identical recovery phase (i.e., acute phase or subacute and/or chronic phase), presumably because the neglect symptoms changed according to the neural reorganization and compensatory strategy during recovery. The present study strongly suggested that the symptomatic features gradually changed over the time course of recovery, even though the patient had an intrinsic/specific brain lesion. This might be one of the reasons why a PC score-based VLSM cannot detect specific lesions (we could only detect brain lesions by VLSM at the uncorrected statistical level). It is quite important that symptoms and neurological behavior not be directly correlated with specific brain areas. Elucidation of the qualitative differences in the changes of symptomatic features (i.e., PC scores and/or assigned clusters) during the recovery process by longitudinal studies is essential when detecting the lesions responsible for the symptomatic features of VSN. We regard this paper as a first step for a future progress, and we are planning to conduct prospective study and then establish rich data for both behavioral measures and brain image in order to overcome the above mentioned limitation of this study in the near future.

### Resource availability

#### Lead contact

Information and requests for resources should be directed to and will be fulfilled by the lead contact. Noritaka Kawashima (nori@rehab.go.jp).

#### Materials availability

N/A.

#### Data and code availability

The data and code used in this study are available on request, in anonymized format, from the corresponding author.

## Methods

All methods can be found in the accompanying Transparent methods supplemental file.

## Supplemental information

Document S1. Transparent methods, Figures S1 and S2, and Tables S1–S3
