# Supplementary material for: Pathological structure of visuospatial neglect: A comprehensive multivariate analysis of spatial and non-spatial aspects
Source: iScience. 2021 Mar 16;24(4):102316. doi: 10.1016/j.isci.2021.102316 (PMC8042346; doi:10.1016/j.isci.2021.102316)
Supplement: Document S1. Transparent methods, Figures S1 and S2, and Tables S1–S3 [file mmc1.pdf]

iScience, Volume *24*

Supplemental information

Pathological structure of visuospatial neglect: A comprehensive multivariate analysis of spatial and non-spatial aspects

Yusaku Takamura, Shintaro Fujii, Satoko Ohmatsu, Shu Morioka, and Noritaka Kawashima

Supplemental Materials

TRANSPARENT METHODS

Participants

Five collaborative hospitals in Japan routinely administer a paper-and-pencil test and touch panel attentional tests (described below) for patients who have suffered a right hemisphere stroke. Both types of tests were taken by 305 patients who were treated at one of these five hospital during the period 2014–2017. For the present cross-sectional study, we retrospectively analyzed the data from the 122 patients who completed both tests and whose detailed data were available. The exclusion criteria were (1) a history of major psychiatric or neurological disorders and (2) the patient found it difficult to understand either test. The average time interval between stroke onset and the testing was 91.23±190.09 days. The mean age of the patients was 66.37±12.08 years. As the clinical routine for evaluating the extent of USN, the patients were scored by the BIT (Wilson et al., 1987) and the Catherine Bergego Scale (CBS; Bergego et al., 1995). In two neuropsychological tests, BIT was conducted for all patients, but CBS was conducted to 49 of 122 patients.

The study procedures and the potential risk of the personal computer (PC)-based evaluation were explained to each patient, and informed consent was obtained from all patients (or a relative of the patient if he or she could not completely understand the study's explanation) before study participation. This study was approved by the Ethics Committee of the National Rehabilitation Center for Persons with Disabilities, Saitama, Japan (reference no. 24-36) and conformed to the tenets of the Declaration of Helsinki.

Evaluation parameters

The patients performed a custom developed reaction time task (@Attention; Creact Corp., Tokyo) using a personal computer with a touch-panel display (21.5 inch), and they completed the Behavioural Inattention Test (BIT) for the evaluation of multiple components of neglect (Fig. 1). The objects of the task are 35 objects (12-mm-dia.) arranged in seven columns and five rows.

Endogenous attention task

The endogenous attention task (EndoAT, Fig. 1A, top) evaluated the patients' 'top-down' endogenous attention. The patient was asked to select all targets in any order (by touching the target on the display with an index finger). When the patient touched a target, the object flashed briefly and then recovered to the default black color. Before the initiation of a trial, the patient was instructed to touch all of the objects and finish when all targets were selected. The propriety of selection, with or without multiple selection (whether a single target was selected more than once), and the selection order (increasing in order from the top left of the display; if the patient could not select the target it was defined as 35 [top right]) were recorded for the later analysis. We quantified each of the six variables for the subsequent analysis (see Fig. 1A and Table 1). We performed the following three steps for the subsequent exploratory/data-driven analysis: a) dimensional reduction from 18 variables using PCA to elucidate the neglect-related deficit components; b) GMM-based probabilistic clustering using the four obtained PCs to elucidate different combinations of neglect-related components for each patient; and c) lesion overlap subtraction of each cluster and VLSM for the four obtained PCs in order to understand the neural mechanisms underlying the neglect-related symptomatic components.

Exogenous attention task

The exogenous attention task (ExoAT, Fig. 1A, middle) evaluated the patients' 'bottom-up' exogenous attention. Based on the randomized function of the software program (LabVIEW ver. 2012, National Instruments, Austin, TX, USA), one of the objects flashed on a 500-ms cycle (black/red alternative switching) until the patient chose it (touched it with an index finger). If the patient could not find the flashing object within 5 seconds, the flashing stopped, and the next object started flashing. Before the initiation of a trial, the patient was instructed to touch the flashing objects as quickly as possible and to not do anything if she/he did not see any flashing object. The propriety of selection and the reaction time required for the choice of each object were recorded in the PC for the later analysis.

Behavioral Inattention Test

The BIT is a conventional test (Fig. 1A, bottom) for evaluations of USN. It includes the following six tests: line cancellation test, letter cancellation test, star cancellation test, copying test, line bisection test, and drawing test. The points on each test were scored by a conventional evaluation method (Ishiai., 1999; Wilson et al.,1987).

Data analysis

Feature extraction based on behavioural test

To visually reveal the spatial distribution of the propriety of selection at both tasks (including reselection and selection order only in the EndoAT) and reaction time on the ExoAT in the 2D plane, we created a space-propriety and time diagram in three dimensions (x = line, y = row, z = propriety and reaction time). Based on the recorded and

scored parameters in both tasks, we quantified a total of 18 variables (Table 1).

The following 6 variables were calculated based on the results of EndoAT: 1) mis-selection count in overall space (MSc_Endo) relating to the general endogenous attention score; 2) the difference of mis-selection count between the left and right space (L-Rdiff_Endo) relating to the neglect in endogenous attention; 3) reselection rate in overall space (%ReSel) relating to the spatial working memory; 4) reselection rate in the left hemi space (%ReSel_L) relating to the spatial working memory in the left space; 5) the initial select position (InitialPos) relating to the attentional bias and exploratory strategy; and 6) the number of midline crossings (CrossMid) relating to the exploratory strategy.

In the same way, we quantified the following 6 variables based on the results of ExoAT: 1) mis-selection count in the overall space (MSc_Exo) relating to the general exogenous attention score; 2) mean reaction time (RTmean), relating to sustained attention and arousal; 3) standard deviation of the reaction time (RTstd), relating to attentional variability and neglect; 4) difference of mis-selection count between the left and right space (L-Rdiff_Exo) relating to neglect in exogenous attention; 5) ratio of the reaction time in the left space to the reaction time in the right space (L/Rratio) relating to neglect in exogenous attention; 6) ratio of the reaction time for the left-end-column target to the reaction time for the right-end-column target (EndlineL/R) relating to neglect in exogenous attention.

In the BIT, the following 6 variables were scored by a conventional evaluation method (Ishiai., 1999; Wilson et al.,1987): 1) the score of the line cancellation test (BIT_Line); 2) the score of the letter cancellation test (BIT_Letter), 3) the score of the star cancellation test (BIT_Star); 4) the score of the line bisection test (BIT_Bisect); 5) the score of the copying test (BIT_Copy); 6) the score of the drawing test (BIT_Draw). In subsequent

analysis, six BIT scores were used with the sign reversed.

All feature extraction processes in EndoAT, ExoAT were performed using MATLAB software and custom scripts (ver. 2015a Mathworks, Natick, MA).

Gaussian mixture model clustering for principal components

The main goal of this study was to classify subtypes of USN consisting of multiple components from the differences in the pathological characteristics in neglect behavior. We thus performed a principal component analysis (PCA) as a common data reduction strategy for obtaining the elements of spatial and non-spatial attention. A PCA is designed to reduce the dimensionality of a data set consisting of many interrelated variables while retaining as much as possible of the variation present in the data set. This is achieved by transforming to a new set of variables, the principal components (PCs), which are uncorrelated, and which are ordered so that the first few PCs retain most of the variation present in all of the original variables. PCA was performed after standardization of all variables. The acceptance criteria of the PCs were defined using a parallel analysis, and the percent of cumulative proportion was >70%.

We then performed Gaussian mixture model (GMM)-based clustering using the acceptable PCs to classify the patients. GMM clustering is a probabilistic model-based clustering method and is more robust than other clustering methods (Banfield and Raftery., 1993). In this clustering approach, the number of clusters and differences in distribution and its volume, shape and orientation can also be compared with statistical information criteria such as the Bayesian information criterion (BIC) and/or integrated the complete-data likelihood (ICL) criterion (Scrucca et al., 2016). We defined the following two criteria for the number of clusters and distribution features: (1) the number of clusters is

3 to 7 (the aim was to provide a more detailed classification than the three categories of severe VSN, moderate USN and no USN), and (2) the model distribution parameter is not equal variance. The model with high theoretical validity with better BIC and ICL values was selected as the optimal model from among several models that met these criteria.

GMM-based clustering has been reported to be useful for characterizing the recovery process on longitudinal evaluations in patients with gait disorders caused by stroke (Dolatabadi et al., 2016). That study applies the computation of cluster membership probabilities for each patient in this probabilistic clustering. In the present study, cases that could also be evaluated longitudinally were fit to the model for a clarification of the recovery process after USN. All clustering procedures were performed by the software program R 3.5.0. We also used the add-on packages of R called psych (Revelle., 2015) and mclust5 (Scrucca., 2016) for a parallel analysis to define the acceptable number of PCs and for the Gaussian mixture model clustering.

Statistical analysis

Differences in basic attributes and the neuropsychological test scores in each cluster were examined with the chi-squared test and Kruskal-Wallis test (post hoc Steel Dwass test). Statistical significance was accepted at $p<0.05$. All statistical procedures were also performed by R 3.5.0.

Brain imaging and lesion analysis

Lesion analyses were performed based on the cranial scans (MRI and CT) in 41 of the 122 patients conducted at their final scan and used for diagnosis. The lesion analyses were performed by experienced brain imaging clinicians (YT, SF, SO). Images were converted

to NIfTI format, and stroke lesions were determined for each patient by drawing the lesion locations directly on the original T2, FLAIR and CT images using MRIcroN software, www.mccauslandcenter.sc.edu/mricro/mricron).

Individual cranial scans and the lesion locations were transferred into stereotaxic space using the normalization algorithm of SPM8 (http://www.fil.ion.ucl.ac.uk/spm) and the Clinical Toolbox (Rorden et al., 2012 (http://www.mricro.com/clinical-toolbox/spm8-scripts). Using the 'MR normalize' algorithm of the Clinical Toolbox, individual cranial scans and lesion locations were transformed to the T1 template based on older individuals with a resampled voxel size of 1 mm^3.

The lesion locations were then compared using the subtraction technique (Rorden and Karnath., 2004) for an investigation of the lesion differences between each cluster. This technique illustrates the nodal point of overlap among lesions associated with a disorder, in direct visual contrast to sites that are not associated with that disorder. The normalized lesion volumes of interest were overlaid on the T1-weighted template MRI-scan from the Montreal Neurological Institute (MNI, http://www.bi.mni.mcgill.ca).

To evaluate the lesion areas with respect to cortical and subcortical gray matter structures, we overlaid the maps on the Automated Anatomical Labelling atlas (Tzourio-Mazoyer et al 2002) distributed with MRIcron. To identify the white matter fiber tracts affected by the lesions, we overlaid the lesion maps with the white matter fiber tract templates from the Johns Hopkins University (JHU) white-matter tractography atlas (Wakana et al., 2007).

RESULTS

Relationships between the BIT and Touch panel-based test (Relevance to Figure 1)

Supplementary figure S1 shows the relationships between the behavioural inattention test and touch panel-based test. As shown in the below scatterplots (Supplementary figure S1B and S1C), the PC1-related variables, RTmean and MSc-Ex, showed strong correlation with BIT. These variables might reflect arousal and attention state. On the other hand, the PC2-related variables, L/Rratio and RTstd, showed weak correlation with BIT, suggesting that the BIT score did not include the exogenous aspect of visuospatial attention. The PC3-related variables, %ReSel, showed moderate correlation with BIT.

Optimal model selection in GMM-based clustering (Relevance to Figures 2 and 4)

Supplementary Table S1 provides the BIC and ICL values of the total of 60 models that met the criteria. For both the BIC and the ICL, Models 1, 2 and 3 were listed as the top three models. From the top three models in which the BIC and ICL are better, six classes with a VEE model (ellipsoidal distribution and variable volume, equal shape, equal orientation) were accepted as models with high theoretical validity.

Details of the lesion subtraction (Relevance to Figure 3)

Detailed results of the subtraction analysis of [Clusters 2–3] and [Cluster (1+2)-3] are listed in Supplementary Table S3. These results correspond to Figure 3 in the main manuscript.

Voxel-based lesion symptom mapping (Relevance to Figure 3)

Supplementary Fig. S2A illustrates the VLSM results regarding the three main

components detected by the PCA. The red-, green- and yellow-highlighted areas indicate statistically significant voxels with the respective PCs (uncorrected $p<0.05$) indicated in the figure. The detected areas of PC1 (arousal and attention state-related) were the insula (x, y, z = 40, −12, 14), inferior frontal gyrus (IFG) (orb: 43, 10, 29, tri: 39, 30, 10), superior longitudinal fasciculus (SLF) (43, −14, 26), angular gyrus (AG) (45, −40, 30), superior temporal gyrus (STG) (46, −24, 15), and supramarginal gyrus (SMG) (50, −22, 26). The exogenous neglect-related PC2 had relevant lesions in the STG (45, −36, 13), middle temporal gyrus (MTG) (39, −48, 13) and SLF (40, −48, 13). The SWM deficit-related PC3 had relevant lesions in the AG (28, −57, 45), middle occipital gyrus (MOG) (31, −62, 39), superior occipital gyrus (SOG) (31, −67, 41), inferior parietal lobule (IPL) (32, −53, 48) and superior parietal lobule (SPL) (32, −55, 49). Supplementary Fig. S2B provides the VLSM results regarding PC4 separately, since this score showed both positive and negative variability, reflecting left or right attention bias. The areas detected for the PC4 left attention bias-related positive score were the MOG (33, −63, 35), AG (44, −70, 35) and SOG (26, −62, 33). The PC4 right attention bias-related negative score had a relevant lesion in the STG (54, −25, 10).

Detailed description of longitudinal cases (Relevance to Figure 5)

Case 1 showed clear stagnation of exogenous attention and remarkable left space neglect in BIT. Even at the fourth measurement (130 days after the onset of stroke), miss-cancellation on the left space remained in all tasks. Since the potential lesions were distributed across a wide range of VAN, we considered that Case 1 could be regarded as having a higher probability of chronicity of visuospatial neglect (Thiebaut de Schotten et al., 2005; Karnath et al., 2011; Lunven et al., 2015).

Case 2 also showed severe attention deficit at his first assessment but showed a gradual improvement in all tasks. However, SWM deficits (such as a higher percentage of re-cancellation in the EndoAT) were observed. Interestingly, the copying and drawing tasks showed an extensive lack of piece in the left space. The persistence of neglect after damage to the parietal cortex is often observed in a clinical context. Therefore, damage to the VAN and parietal cortex may cause neglect symptom due to a failure of different components of spatial attention. The contrast between Case 1 and 2 is a clear example of distinct characteristics/subtypes of severe visuospatial neglect.

Case 3 had a delayed reaction time on the ExoAT, which was presumably due to a lack of arousal and attention state, as reflected by the miss-cancellations. Although Case 3 had a disturbance of visuospatial neglect in the acute phase, this patient showed good recovery at 45 days after the onset of stroke and finally completed both the EndoAT and ExoAT without remarkable errors, and the BIT scores of this patient far exceeded the cut-off at 49 days. Case 3 transited from Cluster 2 to Cluster 4 as a type of rightward bias due to attention deficit.

Case 4 had a delayed reaction time on the ExoAT and showed extensive re-cancellation on the EndoAT. Similar to Case 3, this patient showed good recovery after the onset of stroke and finally completed both the EndoAT and ExoAT without remarkable errors, and the BIT scores of this patient far exceeded the cut-off at 43 days. In contrast to Case 3, Case 4 transited from Cluster 3 to Cluster 5 as a type of compensatory leftward attention. The contrast between Cases 3 and 4 provides a clear example of the distinct characteristics/subtypes of mild visuospatial neglect.

Supplementary Table S1. BIC and ICL in each model, Related to Figure 2 and Figure 4

Bayesian Information Criterion (BIC):

Number of clusters	EEI	VEI	EVI	VVI	EEE	EVE	VEE	VVE	EEV	VEV	EVV	VVV
3 Clusters	1848.16	1613.88	1781.98	1620.40	1783.23	1734.43	1630.03	1588.29	1678.47	1545.14	1676.50	1562.66
4 Clusters	1737.05	1555.66	1755.32	1586.66	1807.01	1772.51	1569.96	1538.77	1721.55	1502.08	1717.77	1513.03
5 Clusters	1693.80	1560.82	1712.09	1598.58	1846.99	1576.44	1575.78	1542.81	1725.14	1532.84	1748.59	1545.85
6 Clusters	1715.64	1530.38	1740.18	1580.18	1627.22	1619.70	1519.95	1528.03	1679.71	1529.61	1712.42	1558.06
7 Clusters	1674.34	1540.32	1719.84	1584.46	1589.86	1671.73	1526.19	1541.99	1726.35	1532.19	1746.78	1599.65

Integrated Complete-data Likelihood (ICL) criterion:

Number of clusters	EEI	VEI	EVI	VVI	EEE	EVE	VEE	VVE	EEV	VEV	EVV	VVV
3 Clusters	1858.57	1622.27	1788.14	1632.94	1788.23	1789.26	1636.51	1592.72	1683.96	1552.85	1688.71	1569.41
4 Clusters	1741.34	1564.91	1798.87	1596.88	1870.36	1887.74	1577.61	1546.32	1777.70	1509.07	1730.05	1521.75
5 Clusters	1702.93	1574.30	1719.38	1608.11	1906.00	1617.32	1588.50	1551.10	1781.92	1538.90	1828.79	1553.87
6 Clusters	1778.93	1537.95	1794.43	1589.62	1635.70	1680.54	1530.25	1534.05	1689.30	1536.12	1786.88	1565.17
7 Clusters	1683.10	1556.44	1728.61	1599.57	1596.31	1753.34	1536.74	1553.16	1756.95	1541.80	1788.13	1612.15

Underlining indicates the top three models.

Abbreviation: EEI: diagonal, equal volume and shape model, VEI: diagonal, varying volume, equal shape, EVI: diagonal, equal volume, varying shape, VVI: diagonal, varying volume and shape, EEE: ellipsoidal, equal volume, shape, and orientation, EVE: ellipsoidal, equal volume and orientation , VEE: ellipsoidal, equal shape and orientation, VVE: ellipsoidal, equal orientation, EEV: ellipsoidal, equal volume and equal shape, VEV: ellipsoidal, equal shape, EVV: ellipsoidal, equal volume, VVV: ellipsoidal, varying volume, shape, and orientation

Supplementary Table S2. Multiple comparisons, Related to Figure 2 and Figure 4

Steel-Dwass			PC1		PC2		PC3		PC4	
Cluster			p-value	r	p-value	r	p-value	r	p-value	r
Cls1	vs.	Cls2	0.015872	0.473	0.005553	0.544	0.999996	0.000	0.883335	0.029
Cls1	vs.	Cls3	0.023877	0.604	0.023877	0.604	0.023877	0.604	0.023877	0.604
Cls1	vs.	Cls4	0.000999	0.440	0.000999	0.440	0.605568	0.069	0.001381	0.427
Cls1	vs.	Cls5	0.003009	0.561	0.003009	0.561	0.003009	0.561	0.012278	0.473
Cls1	vs.	Cls6	0.00539	0.593	0.00539	0.593	0.930924	0.018	0.00539	0.593
Cls2	vs.	Cls3	0.351685	0.176	0.294882	0.198	0.000832	0.632	0.050205	0.370
Cls2	vs.	Cls4	3.96E-09	0.703	0.000273	0.435	0.581807	0.066	0.064736	0.221
Cls2	vs.	Cls5	4.5E-07	0.779	0.005643	0.427	0.005643	0.427	0.00515	0.432
Cls2	vs.	Cls6	5.21E-06	0.759	0.000854	0.556	0.990721	0.002	0.303393	0.172
Cls3	vs.	Cls4	0.007403	0.352	0.793254	0.034	9.48E-05	0.513	0.276959	0.143
Cls3	vs.	Cls5	0.002017	0.564	0.959201	0.009	0.000525	0.633	0.000525	0.633
Cls3	vs.	Cls6	0.001248	0.659	0.064817	0.377	0.001248	0.659	0.104183	0.332
Cls4	vs.	Cls5	0.014007	0.290	0.913258	0.013	3.09E-09	0.698	2.89E-10	0.743
Cls4	vs.	Cls6	4.99E-06	0.562	0.498077	0.083	6.39E-05	0.492	0.991165	0.001
Cls5	vs.	Cls6	0.831127	0.035	0.011953	0.408	2.91E-06	0.759	2.91E-06	0.759

Abbreviation: Cls: Cluster, PC: Principal component

Supplementary Table S3. Details of subtraction analysis, Related to Figure 3

Cluster 1 dominant areas (vs. Cluster 2 and Cluster 3)		
**Area**	**%Overlap**	**Atlas**
Precentral	73%	AAL
Postcentral	68%	AAL
Insula	68%	AAL
Rolandic_Oper	68%	AAL
Frontal_Inf_Oper	62%	AAL
Frontal_Inf_Orb	61%	AAL
SupraMarginal	57%	AAL
Superior_longitudinal_fasciculus	57%	JHU
Frontal_Inf_Tri	56%	AAL
Temporal_Sup	56%	AAL
Heschl	52%	AAL
Frontal_Mid	51%	AAL
Temporal_Mid	51%	AAL
**Cluster 2 dominant areas**		
**Area**	**%Overlap**	**Atlas**
Putamen	71%	AAL
External_capsule	64%	JHU
Insula	64%	AAL
Temporal_Sup	57%	AAL
Superior_longitudinal_fasciculus	51%	JHU
Superior_corona_radiata	51%	JHU
SupraMarginal	50%	AAL
Posterior_corona_radiata	50%	JHU
Frontal_Inf_Oper_R	50%	AAL
Anterior_corona_radiata	50%	JHU
Rolandic_Oper_R	50%	AAL
Heschl_R	50%	AAL
**Cluster 3 dominant areas**		
**Area**	**%Overlap**	**Atlas**
Angular	80%	AAL
Parietal_Sup	80%	AAL
Occipital_Sup	80%	AAL
Temporal_Mid	73%	AAL
Occipital_Mid	73%	AAL
Parietal_Inf	73%	AAL
Superior_longitudinal_fasciculus	66%	JHU
SupraMarginal	66%	AAL
Precuneus	60%	AAL
Temporal_Sup	59%	AAL
Posterior_corona_radiata	53%	JHU
Postcentral	53%	AAL
Cuneus	53%	AAL
Paracentral_Lobule	53%	AAL

Abbreviation: AAL: Automatic anatomical labeling, JHU: Johns Hopkins University white-matter tractography atlas

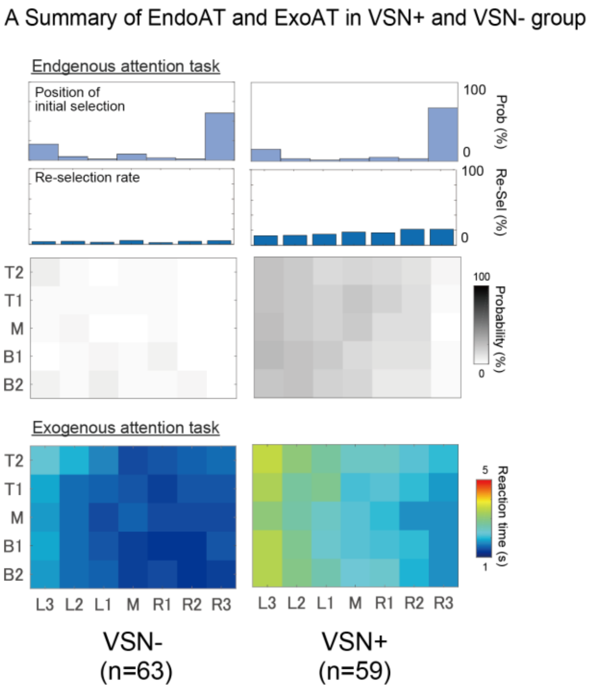

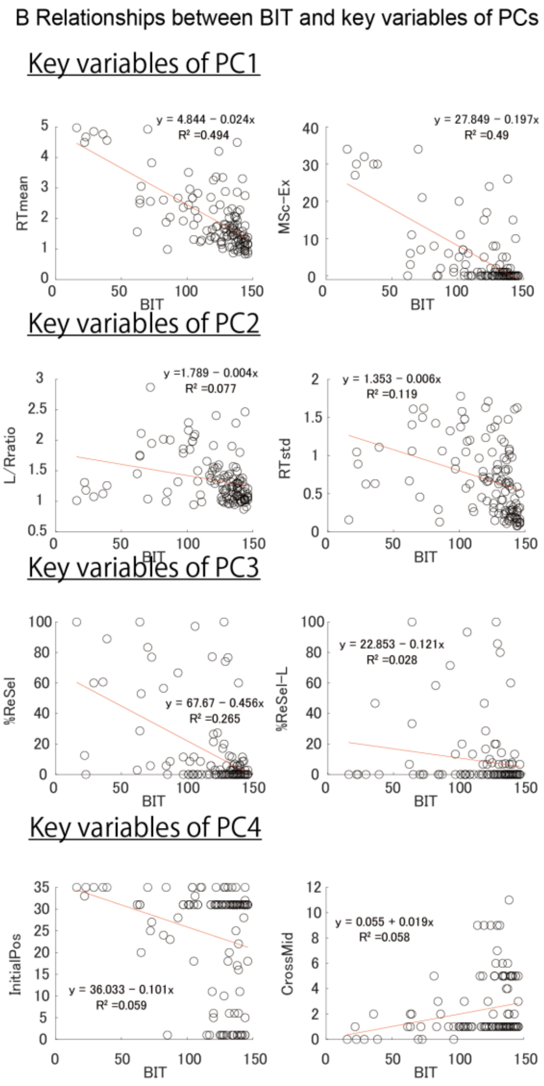

C Correlation analysis

	VarName	r	p
PC1	RTmean	-0.703	1.85E-19
	MSc_Ex	-0.700	2.89E-19
PC2	L/Rratio	-0.277	0.0020031
	RTstd	-0.345	9.89E-05
PC3	%ReSel	-0.514	1.35E-09
	%ReSel_L	-0.168	0.0651279
PC4	InitialPos	-0.242	0.0071518
	CrossMid	0.241	0.0074554

Supplementary Figure S1, Related to Figure 1. A: Summary of EndoAT and ExoAT in patients with VSN+ and VSN+. Presence of VSN was defined using the cut-off score of the behavioural inattention test (BIT). All plots are constructed in a manner similar to that described in Figure 2D. The top row shows the position of the initial selection in EndoAT. The second row shows the reselection rate in EndAT. The third row shows the spatial distribution of the selection probability for each target in EndAT. The bottom row shows the spatial distribution of reaction time in ExoAT. **B**: Scatterplot showing the relationship between BIT and the key variables of four PCs. **C**: Correlation analysis between BIT and the key variables of four PCs.

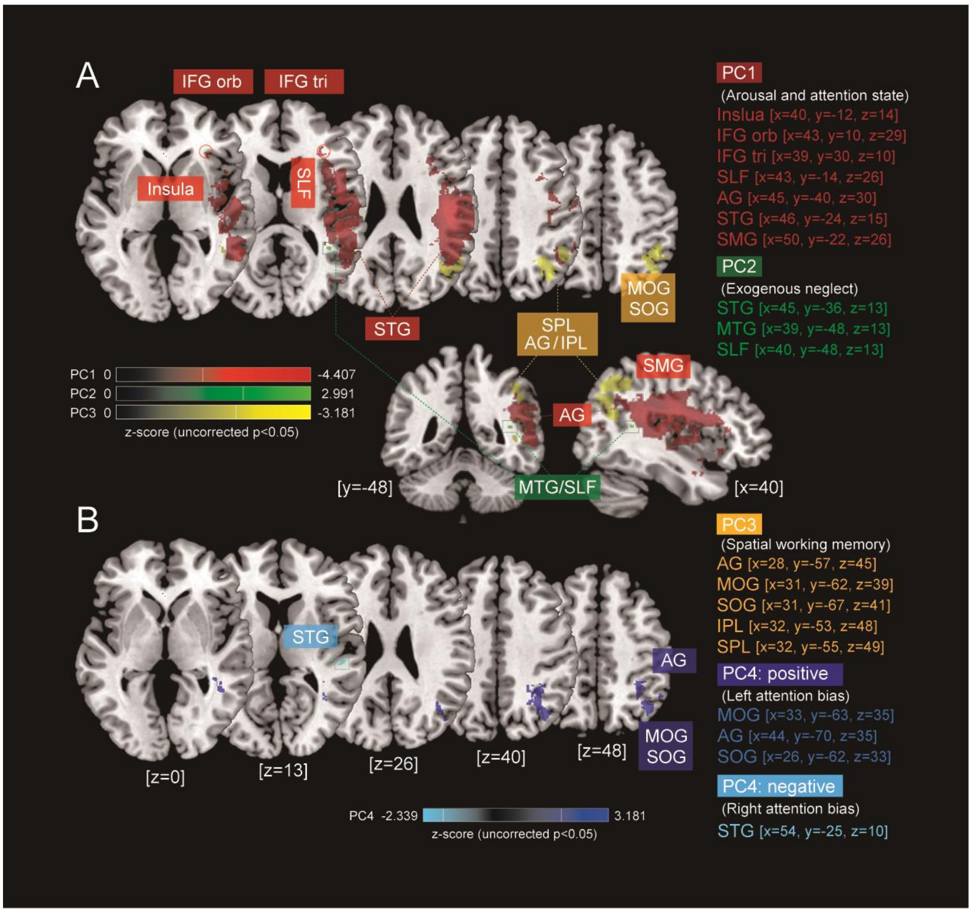

Supplementary Figure S2, Related to Figure 3. The VLSM results regarding the main components detected by the PCA. **A:** Red-, green- and yellow-highlighted areas indicate the voxels significantly correlated with PC1, PC2, and PC3, respectively (uncorrected $p<0.05$). **B:** VLSM results were determined separately for PC4, because the score for this component showed both positive and negative variability, reflecting the left and right attention bias, respectively. The areas highlighted in dark blue and light blue indicate voxels significantly correlated with positive and negative PC4 scores, respectively (uncorrected $p<0.05$).

Supplemental References

Bergego, C., Azouvi, P., Samuel, C., Marchal, F., Louis-Dreyfus, A., Jokic, C., Morin, L., Renard, C., Pradat-Diehl, P., Deloche, G. (1995). Validation d'une échelle d'évaluation fonctionnelle de l'héminégligence dans la vie quotidienne: l'échelle CB. Ann. Readapt. Med. Phys. 38, 183-9.

Dolatabadi, E., Mansfield, A., Patterson, K. K., Taati, B., and Mihailidis, A. (2016). Mixture-model clustering of pathological gait patterns. IEEE J. Biomed. Health. Inf. 21, 1297-1305.

Ishiai, S. (1999). Behavioural Inattention Test. Japanese Version. Sinko Igaku Syuppan, Japan.

Revelle, W., and Revelle, M. W. (2015). Package ‘psych’. The comprehensive r archive network.

Rorden, C., Bonilha, L., Fridriksson, J., Bender, B., and Karnath, H. O. (2012). Age-specific CT and MRI templates for spatial normalization. Neuroimage 61, 957-965.

Rorden, C., and Karnath, H. O. (2004). Using human brain lesions to infer function: a relic from a past era in the fMRI age?. Nat. Rev. Neurosci. 5, 812-819.

Scrucca, L., Fop, M., Murphy, T. B., and Raftery, A. E. (2016). mclust 5: clustering, classification and density estimation using Gaussian finite mixture models. The R journal 8, 289.

Tzourio-Mazoyer, N., Landeau, B., Papathanassiou, D., Crivello, F., Etard, O., Delcroix, N., ... and Joliot, M. (2002). Automated anatomical labeling of activations in SPM using a macroscopic anatomical parcellation of the MNI MRI single-subject brain. Neuroimage 15, 273-289.

Wakana, S., Caprihan, A., Panzenboeck, M. M., Fallon, J. H., Perry, M., Gollub, R. L., ...

and Mori, S. (2007). Reproducibility of quantitative tractography methods applied to cerebral white matter. Neuroimage 36, 630-644.

Wilson, B., Cockburn, J., and Halligan, P. (1987). Development of a behavioral test of visuospatial neglect. Arch. Phys. Med. Rehabil. 68, 98-102.
